# Combination of docetaxel versus nonsteroidal antiandrogen with androgen deprivation therapy for high-volume metastatic hormone-sensitive prostate cancer: a propensity score-matched analysis

**DOI:** 10.1007/s00345-022-04030-2

**Published:** 2022-05-21

**Authors:** Takafumi Yanagisawa, Takahiro Kimura, Kenichi Hata, Shintaro Narita, Shingo Hatakeyama, Keiichiro Mori, Takayuki Sano, Takashi Otsuka, Yuya Iwamoto, Yuki Enei, Minoru Nakazono, Keigo Sakanaka, Kosuke Iwatani, Akihiro Matsukawa, Mahito Atsuta, Hideomi Nishikawa, Shunsuke Tsuzuki, Jun Miki, Tomonori Habuchi, Chikara Ohyama, Shahrokh F. Shariat, Shin Egawa

**Affiliations:** 1 Department of Urology, The Jikei University School of Medicine, 3-19-18, Nishi-shimbashi, Minato-ku, Tokyo, 105-8471 Japan; 2 Department of Urology, Comprehensive Cancer Center, Medical University of Vienna, Vienna, Austria; 3 Department of Urology, Atsugi City Hospital, Kanagawa, Japan; 4 Department of Urology, Akita University School of Medicine, Akita, Japan; 5 Department of Urology, Division of Advanced Blood Purification Therapy, Hirosaki University Graduate School of Medicine, Aomori, Japan; 6 Institute for Urology and Reproductive Health, Sechenov University, Moscow, Russia; 7 Hourani Center for Applied Scientific Research, Al-Ahliyya Amman University, Amman, Jordan; 8 Department of Urology, University of Texas Southwestern Medical Center, Dallas, TX USA; 9 Department of Urology, Second Faculty of Medicine, Charles University, Prague, Czech Republic; 10 Department of Urology, Weill Cornell Medical College, New York, NY USA; 11 Karl Landsteiner Institute of Urology and Andrology, Vienna, Austria

**Keywords:** Metastatic hormone-sensitive prostate cancer, High volume, Docetaxel, Bicalutamide, Nonsteroidal antiandrogen

## Abstract

**Purpose:**

The aim of this study was to investigate the oncologic efficacy of combining docetaxel with androgen deprivation therapy (ADT) versus nonsteroidal antiandrogen (NSAA) with ADT in patients with high-volume metastatic hormone-sensitive prostate cancer (mHSPC) with focus on the effect of sequential therapy in a real-world clinical practice setting.

**Methods:**

The records of 382 patients who harbored high-volume mHSPC, based on the CHAARTED criteria, and had received ADT with either docetaxel (*n* = 92) or NSAA (bicalutamide) (*n* = 290) were retrospectively analyzed. The cohorts were matched by one-to-one propensity scores based on patient demographics. Overall survival (OS), cancer-specific survival (CSS), progression-free survival (PFS), including time to castration-resistant prostate cancer (CRPC), and time to second-line progression (PFS2) were compared. 2nd-line PFS defined as the time from CRPC diagnosis to progression after second-line therapy was also compared.

**Results:**

After matching, a total of 170 patients were retained: 85 patients treated with docetaxel + ADT and 85 patients treated with NSAA + ADT. The median OS and CSS for docetaxel + ADT versus NSAA + ADT were not reached (NR) vs. 49 months (*p* = 0.02) and NR vs. 55 months (*p* = 0.02), respectively. Median time to CRPC and PFS2 in patients treated with docetaxel + ADT was significantly longer compared to those treated with NSAA (22 vs. 12 months; *p* = 0.003 and, NR vs. 28 months; *p* < 0.001, respectively). There was no significant difference in 2nd-line PFS between the two groups.

**Conclusions:**

Our analysis suggested that ADT with docetaxel significantly prolonged OS and CSS owing to a better time to CRPC and PFS2 in comparison to NSAA + ADT in high-volume mHSPC.

**Supplementary Information:**

The online version contains supplementary material available at 10.1007/s00345-022-04030-2.

## Introduction

The management of metastatic hormone-sensitive prostate cancer (mHSPC) has rapidly developed over the last years [1]. For decades, androgen deprivation therapy (ADT), consisting of bilateral orchiectomy or chemical ADT, such as luteinizing hormone-releasing hormone (LHRH) agonists with or without first-generation antiandrogens or antagonists, has been the standard of care for mHSPC [2]. In the last five years, the CHAARTED and STAMPEDE trials revealed that adding docetaxel to ADT significantly improves OS compared to ADT alone, particularly in high-volume mHSPC patients [3, 4]. These findings encouraged “upfront docetaxel” in addition to ADT as a standard treatment for mHSPC in the guidelines [1, 5]. However, ADT alone was not the standard treatment for many practitioners. Indeed, ADT was often combined with first-generation nonsteroidal antiandrogen (NSAA), such as bicalutamide, flutamide, or nilutamide, a strategy widely known as combined or maximum androgen blockade. Since a meta-analysis in 2000 found that bicalutamide with ADT provided only 2–3% of OS benefit over ADT alone in mHSPC patients, oncologic benefit of adding NSAA to ADT has been controversial [6, 7]. Nevertheless, a recent analysis from global database of 6,198 mHSPC patients showed that more than 70% patients in Asia were treated with first-generation NSAA with ADT even from 2018 to 2020 [8]. On the other hand, only the ENZAMET study assessed the NSAA with ADT as a control arm [9]. This has led many clinicians to question of upfront docetaxel with ADT compared to NSAA with ADT in high-volume mHSPC patients. Moreover, while the clinical trials of upfront docetaxel with ADT showed a prolongation in the time to castration-resistant prostate cancer (CRPC) as well as clinical progression, they failed to provide data on the impact on progression-free survival (PFS) after progression to CRPC and time to second-line progression (PFS2) [10, 11]. Indeed, the response to sequential treatment in metastatic CRPC (mCRPC) patients after upfront docetaxel compared to NSAA with ADT is still unclear. Thus, we aimed to clarify the oncologic outcomes, including response to second-line therapy after progression, of upfront docetaxel with ADT compared to NSAA with ADT in high-volume mHSPC patients using a well-described Japanese real-world practice. We adjusted for the effects of potential differences between the cohorts using a propensity score matching.

## Patients and methods

### Patients

Following approval by our institutional review boards (31–478[10060]), we reviewed the records of 382 consecutive patients diagnosed with de novo high-volume mHSPC treated with ADT and either docetaxel (*n* = 92) or NSAA (bicalutamide) (*n* = 290) at 16 hospitals/centers in Japan between September 2015 and December 2020. The definition of high volume was based on the criteria defined in the CHARRTED trial. Patients with high-volume mHSPC were required to have one of the two following risk factors associated with poor prognosis: more than four bone lesions (including at least 1 metastasis outside vertebral column or pelvis) or the presence of measurable visceral metastasis [3].

### Methods

The status of all patients was documented by a positive bone scan or metastatic lesions on computed tomography (CT) or magnetic resonance imaging (MRI) at the time of diagnosis, in accordance with Response Evaluation Criteria in Solid Tumors (RECIST), version 1.1 [12]. The extent of disease (EOD) for bone metastasis was evaluated by a bone scan at diagnosis and defined as follows: EODI: 1 to 5 lesions, II: 6 to 20 lesions, III: more than 20 but less than EOD IV, and IV: generalized uptake, super scan, or more than 75% of axial skeleton [13]. The patients received ADT and either docetaxel (50 mg–75 mg/m^2^ every three or four weeks, maximum six courses) or bicalutamide (80 mg daily), continued until CRPC or incidence of a severe adverse event. Decisions on the dose reduction of initial docetaxel, type of ADT (LHRH agonist, LHRH antagonist, or bilateral orchiectomy), and sequential therapeutic strategy were dependent on the physician’s preference. Primary prophylaxis with granulocyte colony-stimulating factor (G-CSF) was not routinely applied for the patients treated with docetaxel.

### Assessment and follow-up

**Fig. 1:**
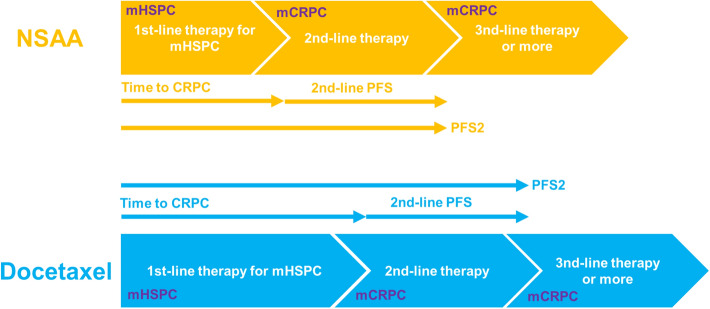
Study schema and definition of outcomes. *NSAA* nonsteroidal antiandrogen, *mHSPC *metastatic hormone-sensitive prostate cancer, *(m)CRPC* (metastatic) castration-resistant prostate cancer, *PFS* progression-free survival

The primary efficacy measure was OS, defined as the time from diagnosis to death from any cause. Secondary measures were as follows: cancer-specific survival (CSS), defined as the time from diagnosis to death from PCa; time to CRPC, defined as the time from diagnosis to develop mCRPC; time to second-line progression (PFS2), defined as the time from diagnosis to progression after second-line therapy [14]; and 2nd-line PFS, defined as the time from CRPC diagnosis to progression after the second-line therapy (Fig. 1).

CRPC was defined as in the European Association of Urology guidelines: castrate serum testosterone < 50 ng/dl or 1.7 nmol/L plus either ① biochemical progression consisting of three consecutive rises in PSA one week apart with two 50% increases over the nadir and PSA > 2 ng/mL, or ② radiological progression consisting of the appearance of new lesions, in the form of either two or more new bone lesions on bone scan or a soft tissue lesion according to the RECIST criteria [5]. Progression after second-line therapy for mCRPC was defined as clinical, biochemical, and/or radiological progression. Biochemical progression was followed the definition of CRPC which is mentioned above.

CT and/or bone scanning was performed every six to twelve months depending on the patient’s condition, PSA, and physician preference. PSA, serum hematologic, and chemical examination were measured at baseline, monthly in the first year, and at least every two months thereafter. Adverse events (AEs) were graded using the Common Terminology Criteria for Adverse Events (CTCAE) of the National Cancer Institute, version 5.0.

### Statistical analysis

Continuous parametric variables are reported as median and interquartile range (IQR). The Chi-square test, Fisher’s exact test, Student’s *t*-test, and Mann–Whitney *U* test were used to compare characteristics of each treatment. Two-sided *p* < 0.05 was considered to be statistically significant. Receiver operating characteristic (ROC) curves were generated for pretreatment PSA, alkaline phosphatase (ALP), lactate dehydrogenase (LDH), and hemoglobin (Hb) values to determine the cut-off values that yielded optimal sensitivity and specificity for the prediction of survival and CRPC (Supplementary Figures. 1, 2). A Cox proportional hazard model was used to analyze prognostic factors for OS and time to CRPC in entire cohorts and each group separately.

Propensity scores were calculated through logistic regression modeling based on the following covariates: age, ALP, Gleason score (GS), Hb, EOD≧III, LDH, type of LHRH, liver metastasis, lung metastasis, lymph node metastasis, PSA at diagnosis, and Eastern Cooperative Oncology Group (ECOG) performance status (PS). Each patient, treated with ADT and either docetaxel or NSAA, was matched 1:1 with the nearest neighbor’s propensity score, using the nearest neighbor matching algorithm without replacement [15]. A caliper size 0.2 times the standard deviation of the logistic regression model of the propensity scores was used to minimize treatment bias [16].

After matching, the Kaplan–Meier method was used to estimate OS, CSS, time to CRPC, 2nd-line PFS, and PFS2. Log-rank tests were used for intertreatment comparisons. All statistical analyses were performed with R version 4.0 (The R Foundation for Statistical Computing, Vienna, Austria).

## Results

### Patient demographics

Data from patient demographics are shown in (Table 1). Prior to matching, the median age at treatment start was significantly lower in the docetaxel group (70 vs. 75 years, *p* < 0.001). Administration of LHRH agonist (43 vs. 60%, *p* = 0.04) and the number of lymph node metastasis (35 vs. 54%, *p* = 0.002) were greater in the NSAA than in the docetaxel group. The propensity score-matched cohorts consisted of 170 patients: 85 in the docetaxel group and 85 in the NSAA group. No statistically significant differences were noted among the measured baseline covariates after propensity score matching.

**Table 1:** Patient demographics

	Before matching			After matching		
	Docetaxel	NSAA	*P* value	Docetaxel	NSAA	*P* value
No. of patients	92	290		85	85	
Age (years), median (IQR)	70 (64–75)	75 (70–80.75)	< 0.001	71 (65–76)	71 (66–76)	0.93
ECOG PS, *n* (%)						
0–1	89 (97)	262 (90)	0.05	82 (96.5)	85 (100)	0.25
≧2	3 (3)	28 (10)		3 (3.5)	0	
PSA at diagnosis (ng/dl), median (IQR)	279.5 (112.6–889.5)	286.1 (71.7–1407)	0.67	265 (118.0–963.2)	244 (71.8–1005)	0.6
Gleason score, *n* (%)						
≦8	24 (26)	76 (26)	0.42	24 (28)	23 (27)	1
≧9	68 (74)	169 (58)		61 (72)	62 (73)	
NA	0	45 (16)				
Extent of disease, *n* (%)						
Lymph nodes	32 (35)	156 (54)	0.002	31 (37)	30 (35)	1
Bone	86 (94)	266 (92)	0.66	80 (94)	79 (93)	1
Lung	27 (29)	80 (28)	0.79	26 (31)	23 (27)	0.74
Liver	5 (5)	12 (4)	0.57	5 (6)	5 (6)	1
Number of bone metastasis, *n* (%)						
≦EODII	47 (51)	160 (55)	0.55	44 (52)	37 (44)	0.36
≧EODIII	45 (49)	130 (45)		41 (48)	48 (56)	
ADT, *n* (%)						
LHRH agonist	40 (43)	175 (60)	0.04	38 (45)	45 (53)	0.74
LHRH antagonist	36 (39)	89 (31)		30 (35)	30 (35)	
Orchiectomy	16 (17)	26 (9)		16 (19)	10 (12)	
Treatment for primary lesion, *n* (%)	7 (8)	13 (4)	0.29	6 (7)	8 (9)	0.78
ALP (IU/l), median (IQR)	374 (237–930.5)	427 (280–926)	0.27	374 (243–948)	412 (268–720)	0.39
LDH (IU/l), median (IQR)	201 (169.8–230.8)	207 (176.5–256)	0.08	201 (170–223)	198 (170–255)	0.62
Hb (g/dl), median (IQR)	13.7 (12.4–14.5)	13.2 (11.7–14.2)	0.02	13.6 (12.4–14.6)	13.6 (12.4–14.6)	0.19

*NSAA* nonsteroidal antiandrogen, *IQR* interquartile range, *ECOG* eastern cooperative oncology group, *PS* performance status, *PSA* prostate-specific antigen, *EOD* extent of disease, *ADT* androgen deprivation therapy, *LHRH* luteinizing hormone-releasing hormone, *ALP* alkaline phosphatase, *LDH* lactate dehydrogenase, *Hb* hemoglobin

※EODI: 1 to 5 lesions, II: 6 to 20 lesions, III: more than 20 but less than EOD IV, IV: generalized uptake, super scan, or more than 75% of axial skeleton

### Analyses of propensity score-matched cohort

#### Oncological outcomes

Median follow-up was 36 months (IQR: 21–46) in the docetaxel group and 28 months (IQR: 16–47) in the NSAA group (Table 2). Median cycles and initial dose of docetaxel were 6 (IQR: 6–6) and 70 mg (IQR: 70–75), and 74 patients (87%) completed planned full dose with six cycles. Progression to CRPC occurred in 55 patients (65%) treated with docetaxel and 63 patients (74%) treated with NSAA. There were 17 deaths in the docetaxel group and 35 in the NSAA group during the follow-up period.

**Table 2:** Treatment details and oncological outcomes

	Docetaxel	NSAA
No. of Patients	85	85
Treatment details		
Median follow-up duration, months (IQR)	36 (21–46)	28 (16–47)
Initial dose (mg), median (IQR)	70 (70–75)	80
Cycles, median (IQR)	6 (6–6)	NA
Achievement of six cycles with full dose, *n* (%)	74 (87)	
Oncological outcomes and sequential treatments		
Nadir PSA (ng/dl), median (IQR)	0.32 (0.05–1.74)	0.44 (0.05–2.16)
Any cause of death, *n* (%)	17 (20)	35 (41)
Cancer-specific death, *n* (%)	15 (18)	32 (38)
Progression to CRPC, *n* (%)	55 (65)	63 (74)
Second-line therapy, *n* (%)		
Abiraterone	35 (71)	17 (32)
Enzalutamide	6 (12)	11 (21)
Docetaxel	0	9 (17)
Cabazitaxel	3 (6)	0
The others (Ra-223/NSAA)	5 (10)	16 (30)

*NSAA* nonsteroidal antiandrogen, *IQR* interquartile range, *CRPC* castration resistance prostate cancer, *PSA* prostate-specific antigen, *NA* not available

**Fig. 2:**
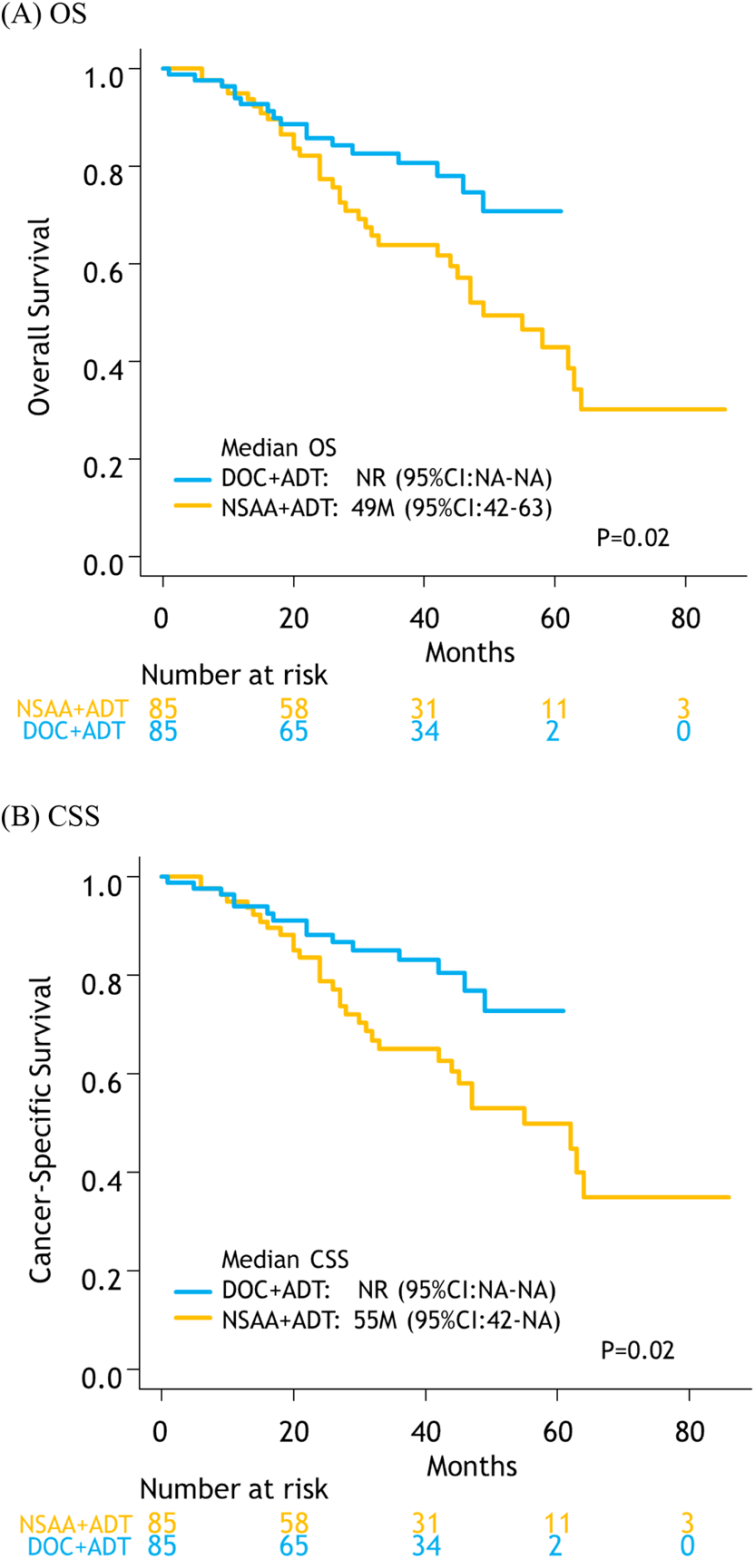
Kaplan–Meier projection of overall **A**, and cancer-specific **B** survival in high-volume mHSPC patients. *NSAA* nonsteroidal antiandrogen, *DOC* docetaxel, *ADT* androgen deprivation therapy, *OS* overall survival; *CSS* cancer-specific survival, *NA* not applicable, *NR* not reached

The median OS was significantly longer in the docetaxel than in the NSAA group (not reached [NR] vs. 49 months [95% CI: 42–63], *p* = 0.02, Fig. 2A). The 3 year OS estimates were 80.7% (95% CI: 69.3–88.2) in the docetaxel and 63.9% (95% CI: 50.9–74.3) in the NSAA group. The median CSS was also significantly longer in the docetaxel than in the NSAA group (NR vs. 55 months [95% CI: 42–not applicable], *p *= 0.02, Fig. 2B). The 3 year CSS estimates were 83.0% (95% CI: 71.8–90.1) in the docetaxel and 65.0% (95% CI: 51.9–75.3) in the NSAA group.

**Fig. 3:**
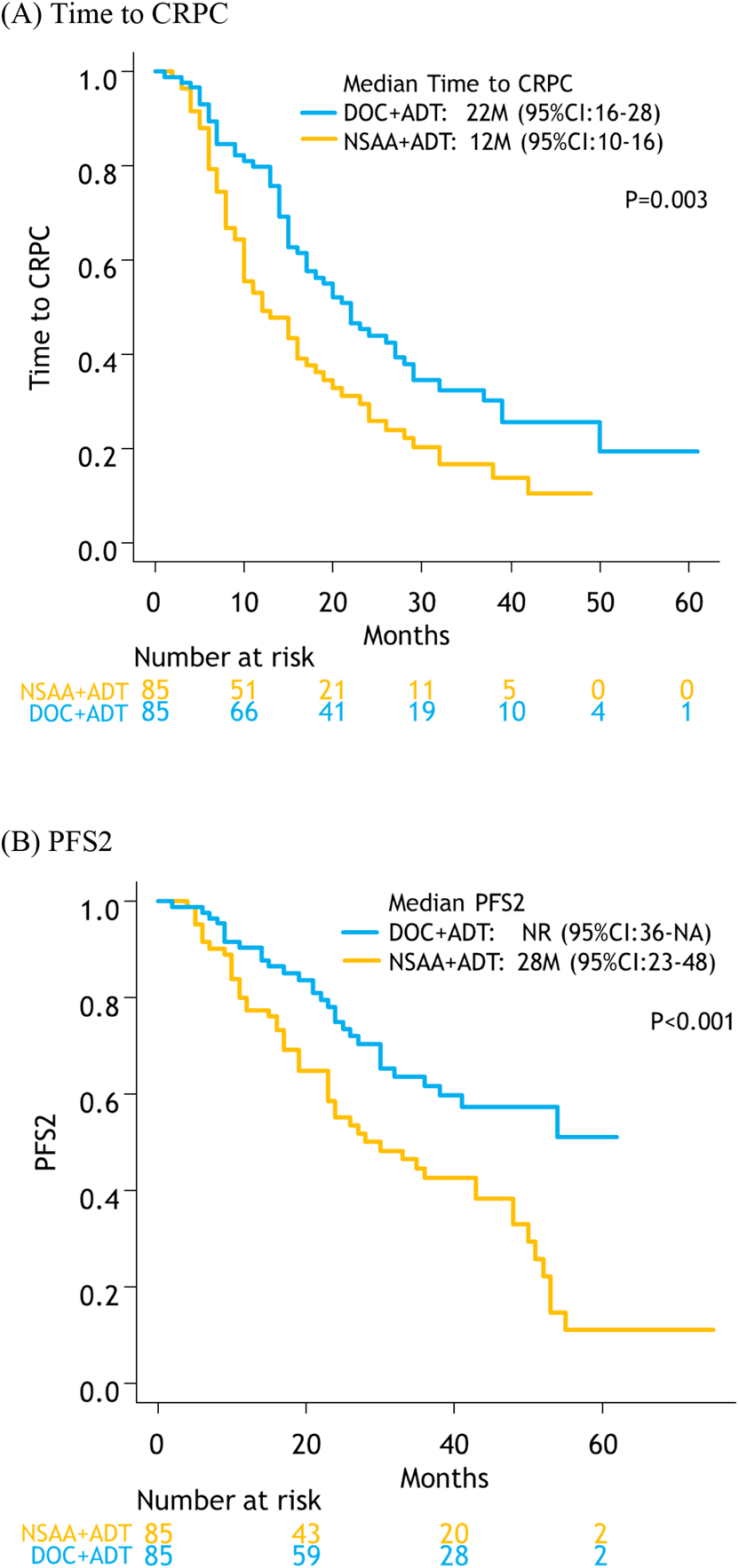
Kaplan–Meier projection of time to CRPC (PFS1) **A**, and time to second-line progression (PFS2) **B** in high-volume mHSPC patients. *NSAA* Nonsteroidal antiandrogen, *DOC* docetaxel, *ADT* androgen deprivation therapy, *CRPC* castration-resistant prostate *Cancer*, *PFS* progression-free survival, *NA* not applicable, *NR* not reached

Median time to CRPC was significantly longer in the docetaxel than in the NSAA group (22 [95% CI: 16–28] vs. 12 months [95% CI: 10–16], *p *= 0.003, Fig. 3A). Median PFS2 was significantly longer in the docetaxel (NR [95% CI: 36–NA]) than in the NSAA group (28 months [95% CI: 23–48]) (*p *< 0.001, Fig. 3B).

**Fig. 4:**
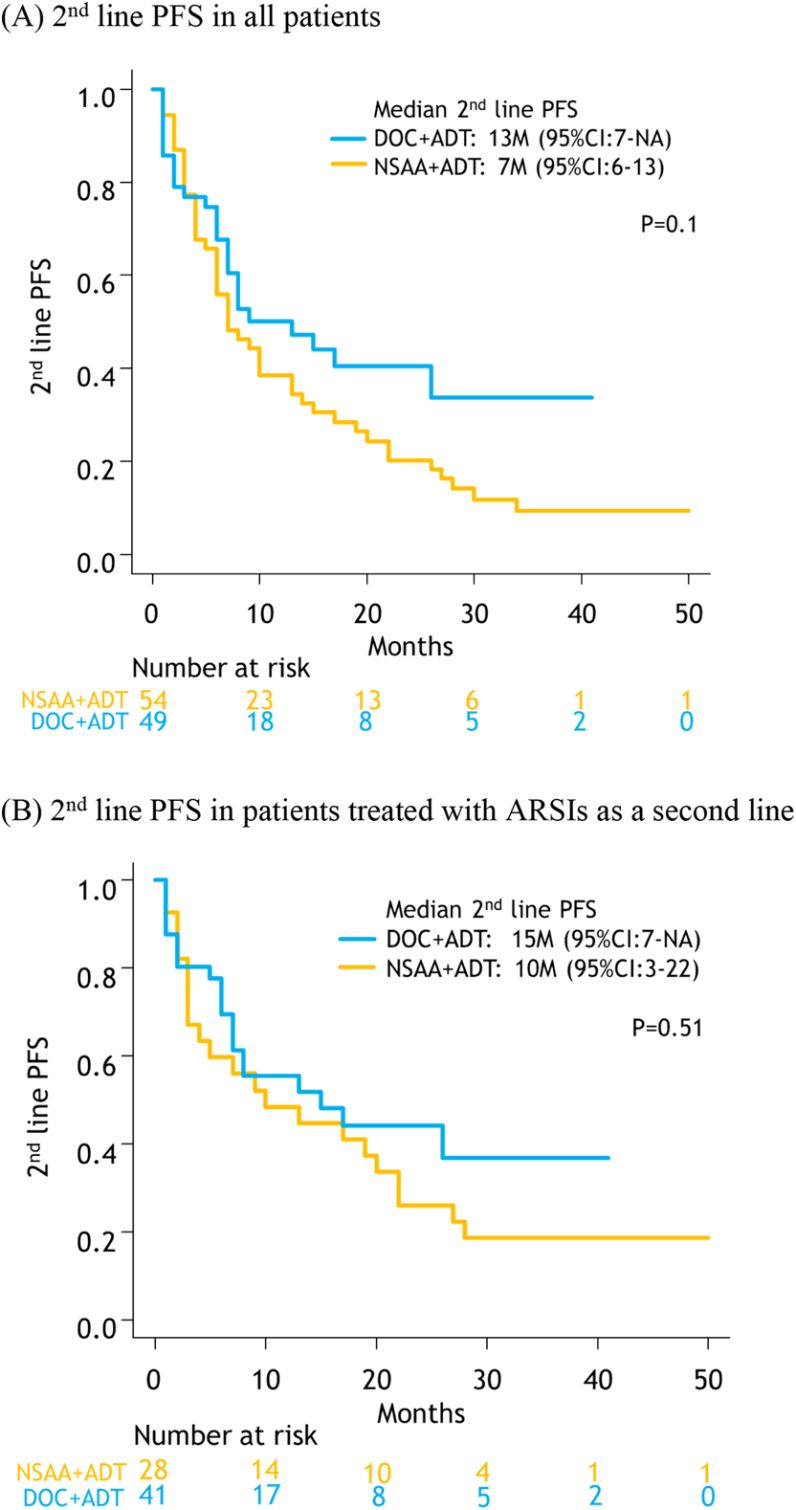
Kaplan–Meier projection of second-line progression-free survival in high-volume mHSPC patients; overall (**A**), subgroup analysis in patients treated with ARSIs as a second line (**B**). *NSAA* nonsteroidal antiandrogen, *DOC* docetaxel, *ADT* androgen deprivation therapy, *ARSI* androgen receptor signaling inhibitor, *NA* not applicable

Forty-nine patients underwent second-line therapy in the docetaxel versus 54 in the NSAA group (Table 2). There was no significant difference in 2nd-line PFS between the two groups (docetaxel: 13 months [95% CI: 7–NA] vs. NSAA: 7 months [95% CI: 6–13], *p* = 0.1, Fig. 4A). Among these patients, subgroup analysis of 2nd-line PFS only in patients treated with androgen receptor signaling inhibitors (ARSIs), such as abiraterone or enzalutamide, showed that there was no significant difference between the two groups (docetaxel: 15 months [95% CI: 7–NA] vs. NSAA: 10 months [95% CI: 3–22], *p* = 0.5, Fig. 4B).

#### Adverse events

Table 3 shows the results of AEs in both treatment groups. Overall AEs were observed in 79% of patients in the docetaxel group and 35% in the CAB groups (*p *< 0.001). Severe AEs, defined as CTCAE grade 3 or more, were observed in 53% of patients in the docetaxel group and 1% in the CAB group (*p *< 0.001). Regarding docetaxel-related AEs, most of the severe AEs were febrile neutropenia (FN) (8%) and neutropenia (49%). One patient who had massive liver metastasis died from liver rupture during the course of docetaxel.

**Table 3:** Treatment details and adverse events of docetaxel compared to bicalutamide

	Docetaxel		NSAA	*P* value
Primary prophylaxis with G-CSF, *n* (%)	20 (24)		NA	
Adverse events, *n* (%)				
Any AEs	67 (79)		30 (35)	< 0.001
Severe AEs (CTCAE Grade ≧ 3)	45 (53)		1 (1)	< 0.001
Any AEs leading to death	1		0	1
Details of docetaxel-related AEs, n (%)	≦Grade 2	Grade 3≦		
Febrile neutropenia	NA	7 (8)		
Neutropenia	8 (9)	42 (49)		
Peripheral neuropathy	17 (20)	0		
Edematous	5 (6)	0		
Dysgeusia	20 (24)	0		
Alopecia	20 (24)	0		

*NSAA* nonsteroidal antiandrogen, *NA* not applicable, *IQR* interquartile range, *G-CSF* granulocyte colony-stimulating factor, *AE* adverse events, *CTCAE* common terminology criteria for adverse events

### Prognostic factors of overall survival and time to CRPC

Tables 4, 5 show the results of univariable and multivariable analyses using a Cox proportional hazard model for prognostic factors of OS. Among all cohorts, in multivariable analysis, GS≧9, LDH≧223, Hb < 12.4, and administration of NSAA were all independent prognostic factors of shorter OS. In the docetaxel group, PS≧2, LDH≧223, and liver metastasis were independent prognostic factors of shorter OS. On the other hand, GS≧9, LDH≧223, and Hb < 12.4 were independent prognostic factors of shorter OS in the NSAA group.

**Table 4:** Impact of possible prognostic factors on overall survival in entire cohorts

	Univariable	*P* value	Multivariable	*P* value
	HR (95% CI)		HR (95% CI)	
Age≧75	1.71 (1.21–2.42)	0.002	1.30 (0.87–1.96)	0.2
PS≧2	1.41 (0.76–2.62)	0.28		
PSA≧654	1.21 (0.86–1.72)	0.28		
Gleason score≧9	3.20 (1.85–5.53)	< 0.001	2.82 (1.62–4.90)	< 0.001
Number of bone metastasis (EOD≧III)	1.72 (1.21–2.43)	0.002	1.05 (0.68–1.62)	0.84
ALP≧429	2.06 (1.43–2.96)	< 0.001	1.40 (0.94–2.10)	0.1
LDH≧223	2.68 (1.89–3.8)	< 0.001	2.52 (1.71–3.71)	< 0.001
Hb < 12.4	2.48 (1.75–3.51)	< 0.001	2.03 (1.37–2.99)	< 0.001
Lung metastasis	0.89 (0.60–1.34)	0.58		
Liver metastasis	2.28 (1.11–4.69)	0.02	1.97 (0.85–4.55)	0.11
LHRH agonist vs. antagonist	1.05 (0.71–1.55)	0.8		
NSAA vs. docetaxel	2.02 (1.22–3.33)	0.006	1.71 (1.02–2.87)	0.04

*HR* hazard ratio, *CI* confidential intervals, *PS* performance status, *PSA* prostate-specific antigen, *EOD* extent of disease, *ALP* alkaline phosphatase, *LDH* lactate dehydrogenase, *Hb* hemoglobin, *LHRH* luteinizing hormone-releasing hormone, *NSAA* nonsteroidal antiandrogen

※EODI: 1 to 5 lesions, II: 6 to 20 lesions, III: more than 20 but less than EOD IV, IV: generalized uptake, super scan, or more than 75% of axial skeleton

**Table 5:** Differential impact of possible prognostic factors on overall survival between NSAA + ADT and DOC + ADT

	Docetaxel				NSAA			
	Univariable		Multivariable		Univariable		Multivariable	
	HR (95% CI)	*P* value	HR (95% CI)	*P* value	HR (95% CI)	*P* value	HR (95% CI)	*P* value
Age≧75	0.97 (0.35–2.72)	0.95			1.74 (1.19–2.54)	0.004	1.39 (0.88–2.20)	0.16
PS≧2	4.07 (0.93–17.9)	0.06	5.35 (1.00–29.2)	0.05	1.14 (0.57–2.25)	0.71	0.7 (0.31–1.58)	0.39
PSA≧654	2.04 (0.81–5.15)	0.13	0.74 (0.25–2.23)	0.6	1.10 (0.76–1.61)	0.61		
Gleason score≧9	1.32 (0.43–4.02)	0.62	0.68 (0.19–2.38)	0.55	4.05 (2.15–7.62)	<0.001	3.67 (1.94–6.94)	<0.001
Number of bone metastasis (EOD≧III)	3.56 (1.26–10.0)	0.02	1.65 (0.40–6.81)	0.49	1.51 (1.04–2.19)	0.03	0.92 (0.58–1.47)	0.74
ALP≧429	3.42 (1.28–9.12)	0.01	2.78 (0.89–8.71)	0.08	1.78 (1.20–2.62)	0.004	1.32 (0.86–2.05)	0.21
LDH≧223	6.38 (2.38–17.1)	< 0.001	6.09 (2.26–16.4)	< 0.001	2.18 (1.5–3.18)	< 0.001	2.17 (1.42–3.30)	< 0.001
Hb < 12.4	2.42 (0.90–6.52)	0.08	1.82 (0.59–5.59)	0.3	2.31 (1.59–3.36)	< 0.001	2.13 (1.40–3.26)	< 0.001
Lung metastasis	1.25 (0.47–3.34)	0.65			0.85 (0.54–1.33)	0.47		
Liver metastasis	5.95 (1.68–21.1)	0.006	5.28 (1.45–19.3)	0.01	1.60 (0.65–3.92)	0.31	1.18 (0.37–3.81)	0.78
LHRH agonist vs. antagonist	1.14 (0.62–2.08)	0.68			0.92 (0.61–1.40)	0.71		

*HR* hazard ratio, *CI* confidential intervals, *PS* performance status, *PSA* prostate-specific antigen, *EOD* extent of disease, *ALP* alkaline phosphatase, *LDH* lactate dehydrogenase, *Hb* hemoglobin, *LHRH* luteinizing hormone-releasing hormone, *NSAA* nonsteroidal antiandrogen

※EODI: 1 to 5 lesions, II: 6 to 20 lesions, III: more than 20 but less than EOD IV, IV: generalized uptake, super scan, or more than 75% of axial skeleton

For time to CRPC, Supplementary Table 1 and Supplementary Table 2 show the results of univariable and multivariable analyses using a Cox proportional hazard model for prognostic factors of time to CRPC. Among all cohorts, in multivariable analysis, GS≧9, ALP≧514, LDH≧227, Hb < 12.2, liver metastasis, and administration of NSAA were all independent prognostic factors of shorter time to CRPC. In the docetaxel group, ALP≧514 and Hb < 12.2 were independent prognostic factors of shorter time to CRPC. On the other hand, GS≧9, ALP≧514, LDH≧227, and Hb < 12.2 were independent prognostic factors of shorter time to CRPC in the NSAA group.

## Discussion

We found that upfront docetaxel with ADT for high-volume mHSPC patients was associated with significantly better OS and CSS in comparison with NSAA with ADT in real-world practice. For all mHSPC cohorts, long-term survival analyses from the CHAARTED trial and the STAMPEDE trial showed that adding docetaxel to ADT significantly improves OS compared to ADT alone [10, 11]. For high-volume mHSPC patients, long-term survival analysis from the CHAARTED trial also reported a significant median OS benefit of 16.8 months in favor of upfront docetaxel (median OS: 51.2 vs. 34.4 months, HR:0.63 [95% CI:0.50–0.79], *p* < 0.001) [11]. Another long-term survival analysis from the STAMPEDE trial reported that the median OS was 39.9 months in favor of upfront docetaxel compared with 35.2 months for ADT alone in high-volume mHSPC patients, but not statistically significant (HR:0.81 [95% CI:0.64–1.02], *p* = 0.064) [10]. In addition, the GETUG-AFU15 trial failed to find a statistical significance with a median OS in high-volume mHSPC patients was 39.8 months for docetaxel versus 35.1 months for patients treated with ADT alone (HR:0.78 [95% CI:0.56–1.09], *p* = 0.14) [17]. In the present study, the median and the 3 year OS were NR and 80.7% for patients treated with docetaxel plus ADT, compared to 49 months and 63.9% for those treated with NSAA plus ADT. Even though the median OS for NSAA with ADT in our study was longer than that for ADT alone in previous RCTs [10, 11, 17], docetaxel improved OS beyond NSAA when given upfront with ADT for high-volume mHSPC. A previous real-world data study reported the clinical utility of upfront docetaxel with focus on PSA-PFS or time to CRPC using matched cohorts [18]; however, this is the first report showing the benefit of OS and CSS for upfront docetaxel in real-world practice with matched cohorts.

We found that treatment with upfront docetaxel plus ADT for high-volume mHSPC was associated with significantly better PFS2 compared to NSAA plus ADT. Among three major RCTs, there were no data regarding PFS2 in patients treated with upfront docetaxel plus ADT [10, 11, 17]. In this study setting, PFS2 could be dependent on time to CRPC and second-line PFS. Our analysis showed that the median time to CRPC for upfront docetaxel with ADT was significantly longer compared to NSAA with ADT (22 vs. 12 months, *p* = 0.003). However, there was no significant difference in second-line PFS between the two groups, even when limited to patients treated with ARSIs for 2nd-line therapy. Francini et al. reported that the efficacy of ARSIs as a sequential therapy after progression was similar regardless of previous use of docetaxel in a total of 102 mHSPC patients [19]. On the other hand, Tsaur et al. reported that a longer time to CRPC predicts more favorable PFS2 in a real-world setting of 65 mHSPC patients who experienced progression after upfront docetaxel [20]. Martini et al. reported that progression to CRPC within six months was the best surrogate for predicting OS in patients with mHSPC using the cohort of the CHAARTED trial [21]. Taken together, in high-volume mHSPC patients, treatment with docetaxel plus ADT leads to longer time to CRPC as well as better PFS2, OS, and CSS, compared to NSAA plus ADT.

The clinical importance of prolonging the time to CRPC has been demonstrated previously even before the era of upfront intensification of treatment [22, 23]. Frees et al. reported that time to CRPC is a significant prognosticator of OS [22]. Miyake et al. reported that mHSPC patients with longer time to CRPC were likely to achieve a more favorable OS, since time to OS after CRPC was similar regardless of time to CRPC [23]. In addition, Hatakeyama et al. reported that time to CRPC was significantly different between the low- and high-volume disease groups, but there was no difference between them in time to OS after CRPC [24]. Therefore, time to CRPC ought to be prolonged as much as possible. In the present study, as we found that 2nd-line PFS was similar in both groups, the clinical importance of prolonged time to CRPC is upheld.

We found that GS≧9, high LDH, low Hb, and administration of NSAA were independent prognostic factors of shorter OS in patients with high-volume mHSPC patients. Hematologic and chemical markers, such as Hb [25], ALP [26], and LDH [27], are known to be prognostic of mortality and progression in patients with metastatic CRPC.

Regarding the prognosticators of time to CRPC in mHSPC patients, we previously have shown that GS≧9, high ALP, and high LDH were independent prognostic factors of worse time to CRPC in high-risk mHSPC patients; we found that upfront abiraterone with ADT prolonged the time to CRPC in patients with all these risk factors [28]. In line with these findings, we found that GS≧9, ALP≧514, LDH≧227, and administration of NSAA were all independent prognostic factors of shorter time to CRPC in all cohorts. Hb < 12.2 and liver metastasis were also prognosticators for worse time to CRPC. Furthermore, our subgroup analyses detected the differential prognosticators for OS and time to CRPC between the docetaxel and the NSAA group (Table 5, Supplementary Table 2). Our findings suggest that GS≧9 seems to be a reliable prognosticator for shorter time to CRPC and OS in the NSAA group, but not in the docetaxel group. These findings might help guide the patient selection for upfront docetaxel; however, further investigations are needed to select the optimal candidates who are most likely to benefit from upfront intensification therapy, helping clinical decision-making.

AEs, such as febrile neutropenia, which is life-threatening AE following docetaxel, affect the optimal treatment selection for mHSPC. In the CHARRTED trial, there was 6.1% for FN and 12.1% for neutropenia greater than CTCAE Grade3 [3]. In the present study, FN was observed in 8% in line with the CHARRTED trial; however, severe neutropenia (CTCAE≧Grade3) was observed in 49% more than the CHARRTED trial despite the primary prophylaxis with G-CSF in some patients. Japanese patients have been shown to be likely to develop severe neutropenia, which was reported as high as 93% in a phase 2 study of docetaxel for mCRPC [29]. Therefore, our results of hematologic AEs lack generalizability. However, our findings highlight the importance of adequate assessment, prevention, and treatment for neutropenia when docetaxel is applied for mHSPC patients.

The present study suffers from several limitations that need to be taken into account. First, it is a retrospective cohort study with a limited number of patients due to the propensity score matching. Thus, only a limited number of patients developed CRPC and received second-line treatment, making the number and demographics of patients who received second-line treatment unmatched. Second, sequential therapy after progression in the NSAA group included 14 patients treated with flutamide which is not currently guideline endorsement treatment. Therefore, we conducted subgroup analysis of 2nd-line PFS in only patients treated with ARSIs as a second line. However, this limitation might lead to underestimating the survival outcomes of the NSAA group. Third, the dosage of bicalutamide (80 mg daily), which has been only approved in Japan, is higher than that in other countries. Fourth, despite using propensity scores, patient demographics were not wholly matched between the two groups; this does not make up for randomization. Finally, ARSIs or docetaxel combined with ADT provided a significant survival benefit in phase 3 RCTs [4, 11, 30–32]; however, there is currently no clear consensus on their comparative effectiveness/tolerability efficacy and predictive biomarkers remain to be standardized.

Despite these limitations, the present study of Japanese real-world data first demonstrated the clinical utility of ADT with docetaxel compared to NSAA in high-volume mHSPC patients.

## Conclusions

Using a Japanese real-world practice setting, we could show that docetaxel with ADT prolonged OS and CSS, owing to a better time to CRPC and PFS2 compared to NSAA with ADT in patients with high-volume mHSPC. In addition, GS≧9, low level of pretreatment Hb, high level of pretreatment LDH, and administration of NSAA were prognostic factors of poor OS in patients with high-volume mHSPC.

## Supplementary Information

Below is the link to the electronic supplementary material.

Supplementary file1 (DOCX 546 KB)

## Data Availability

Not applicable.

## Abbreviations

ADT: Androgen deprivation therapy
ALP: Alkaline phosphatase
ARSI: Androgen receptor signaling inhibitor
CSS: Cancer-specific survival
CT: Computed tomography
CTCAE: Common terminology criteria for adverse events
CRPC: Castration-resistant prostate cancer
ECOG: Eastern cooperative oncology group
EOD: Extent of disease
G-CSF: Granulocyte colony-stimulating factor
GS: Gleason score
Hb: Hemoglobin
LDH: Lactate dehydrogenase
LHRH: Luteinizing hormone-releasing hormone
IQR: Interquartile range
mHSPC: Metastatic hormone-sensitive prostate cancer
MRI: Magnetic resonance imaging
NA: Not applicable
NR: Not reached
NSAA: Nonsteroidal antiandrogen
OS: Overall survival
PFS: Progression-free survival
PS: Performance status
PSA: Prostate-specific antigen
RECIST: Response evaluation criteria in solid tumors
RCTs: Randomized control trials
ROC: Receiver operating characteristic (curve)
