# Supplementary material for: Combination of docetaxel versus nonsteroidal antiandrogen with androgen deprivation therapy for high-volume metastatic hormone-sensitive prostate cancer: a propensity score-matched analysis
Source: World J Urol. 2022 May 21;41(8):2051–62. doi: 10.1007/s00345-022-04030-2 (PMC10415473; doi:10.1007/s00345-022-04030-2)
Supplement: Supplementary file 1 — Supplementary file1 (DOCX 546 KB) [file 345_2022_4030_MOESM1_ESM.docx]

**Supplementary Information**

Supplementary Figure.1: Receiver operating characteristic (ROC) curves to assess the optimal cut-off values of (A) serum prostate specific antigen (PSA), (B) alkaline phosphatase (ALP), (C) hemoglobin (Hb), and (D) lactate dehydrogenase (LDH) for overall survival (OS)

(A) PSA (B) ALP


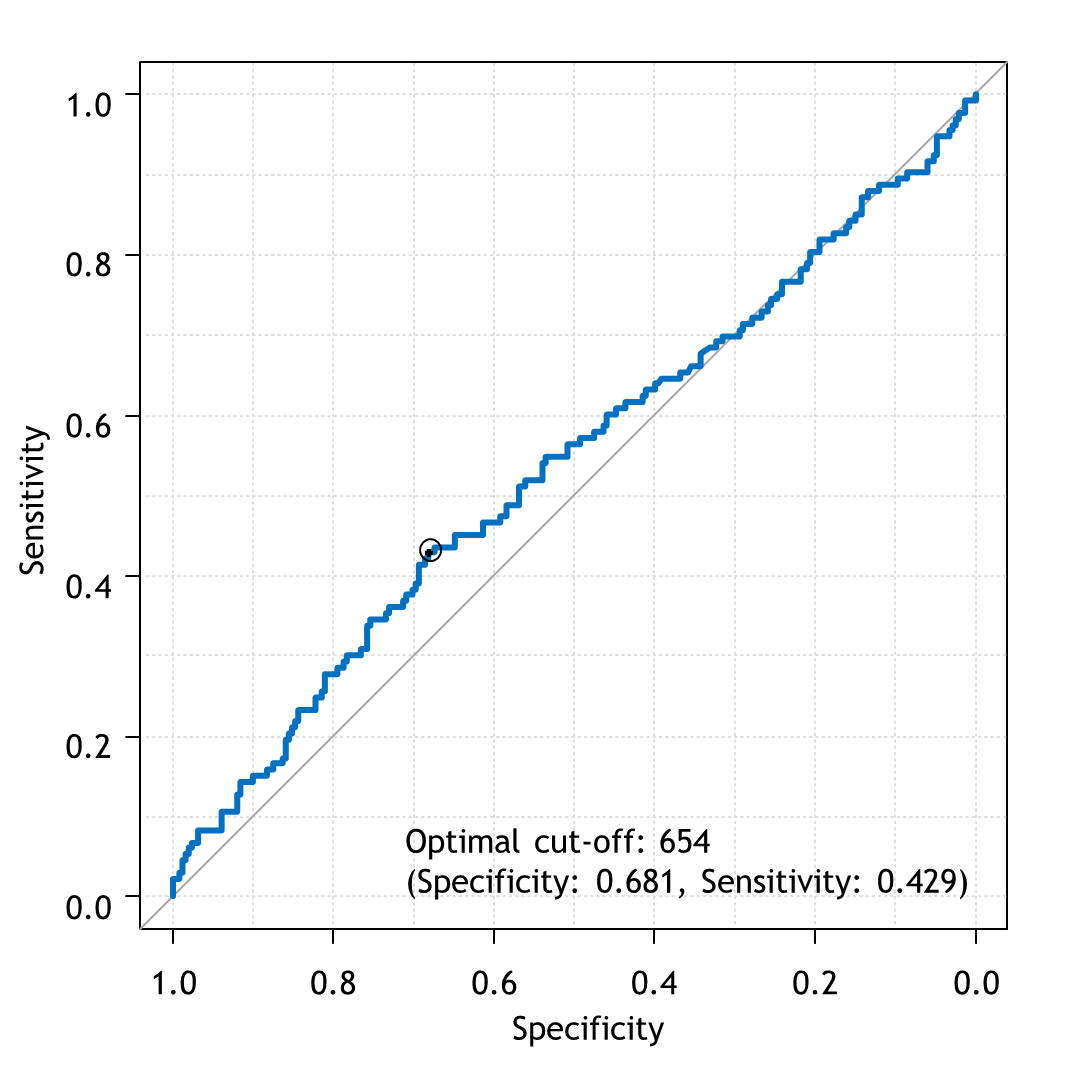

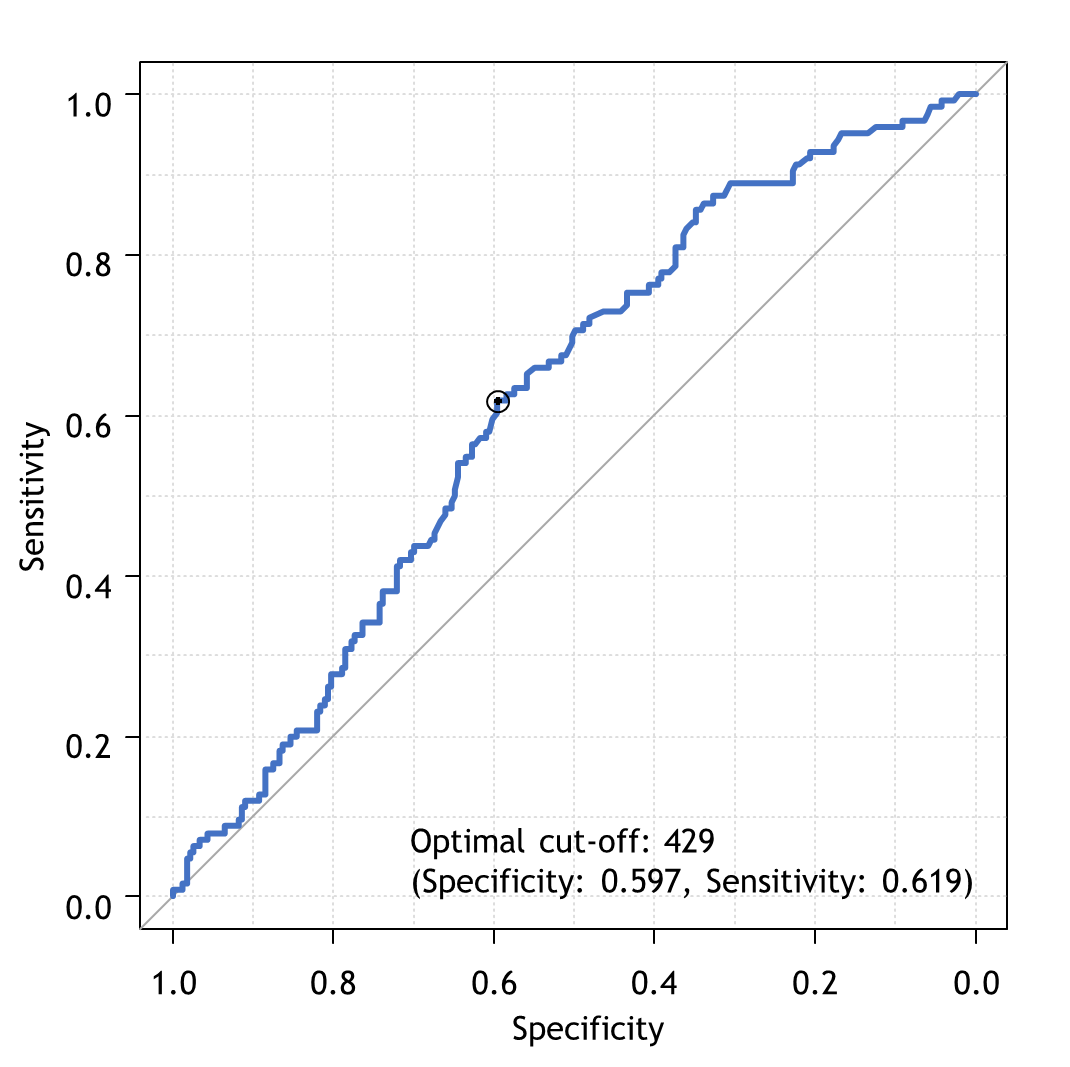


(C) Hb (D) LDH


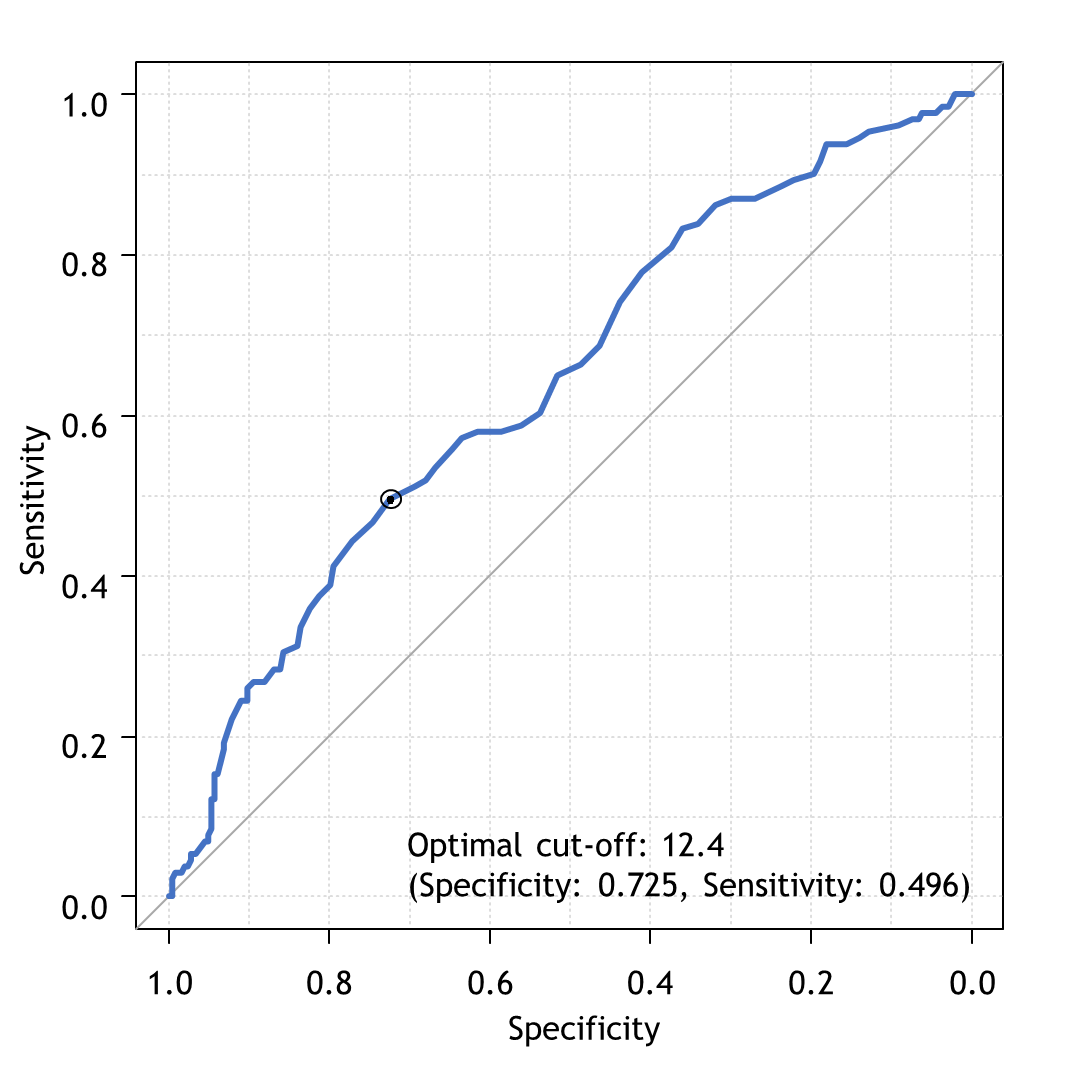

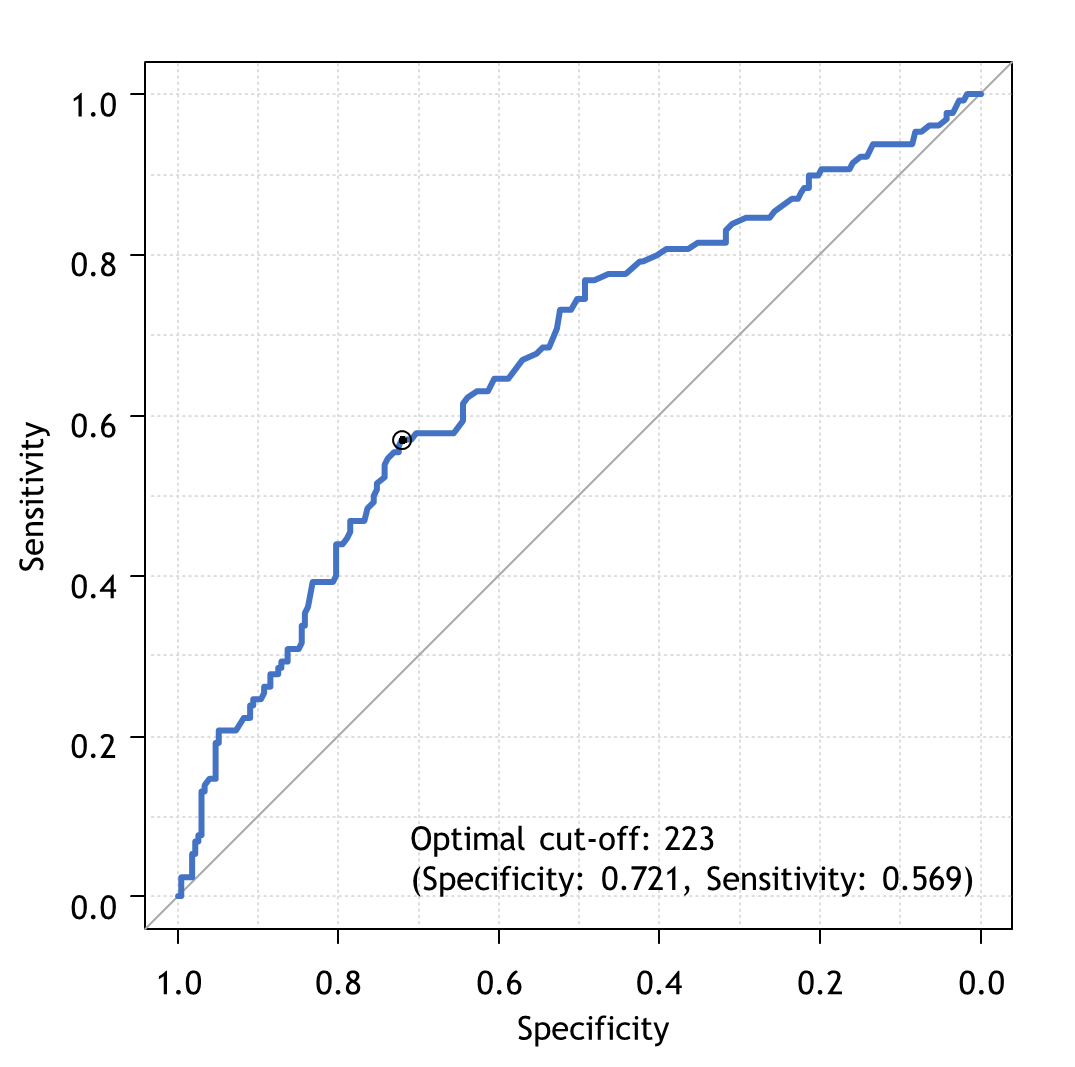


Supplementary Figure.2: Receiver operating characteristic (ROC) curves to assess the optimal cut-off values of (A) serum prostate specific antigen (PSA), (B) alkaline phosphatase (ALP), (C) hemoglobin (Hb), and (D) lactate dehydrogenase (LDH) for time to CRPC

(A) PSA (B) ALP


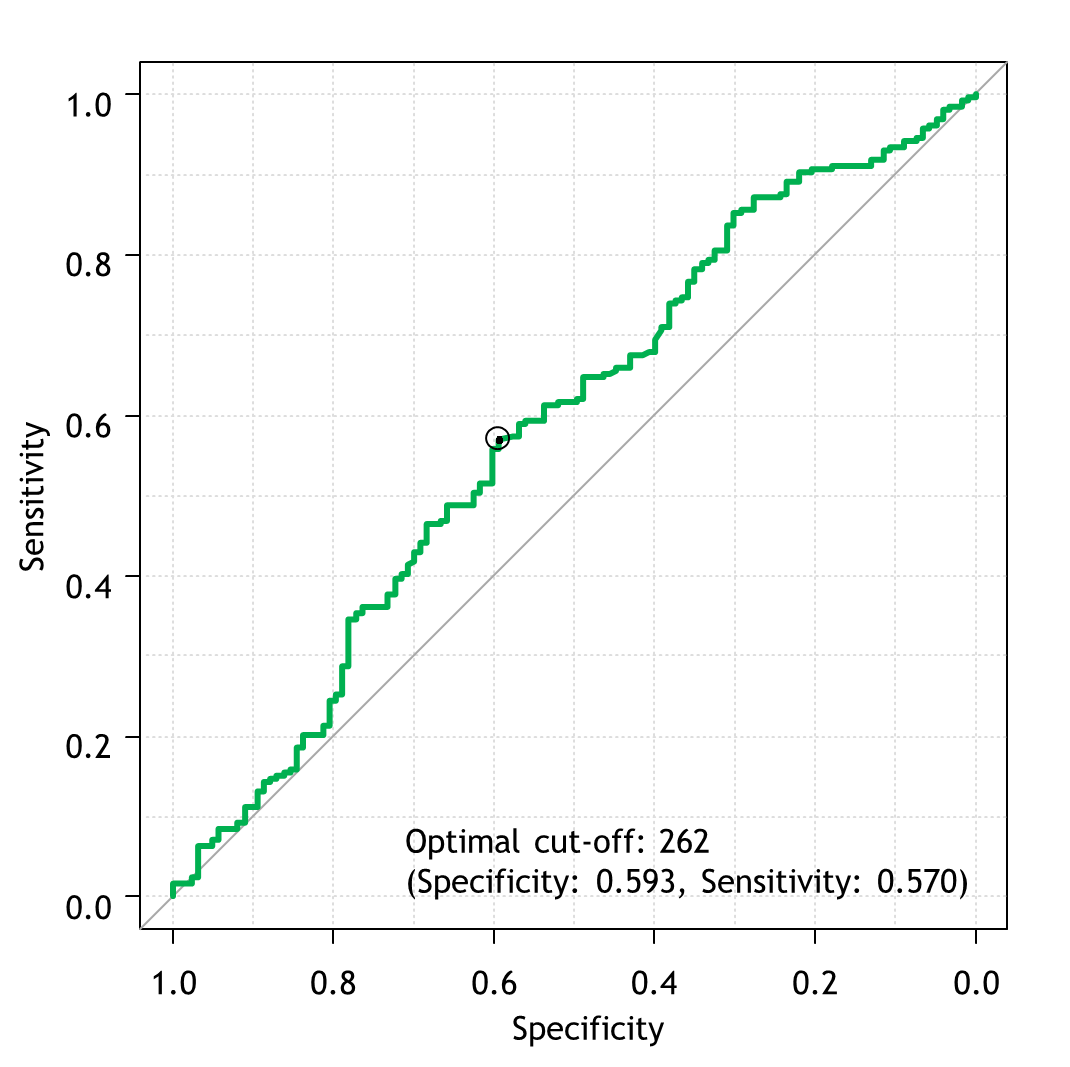

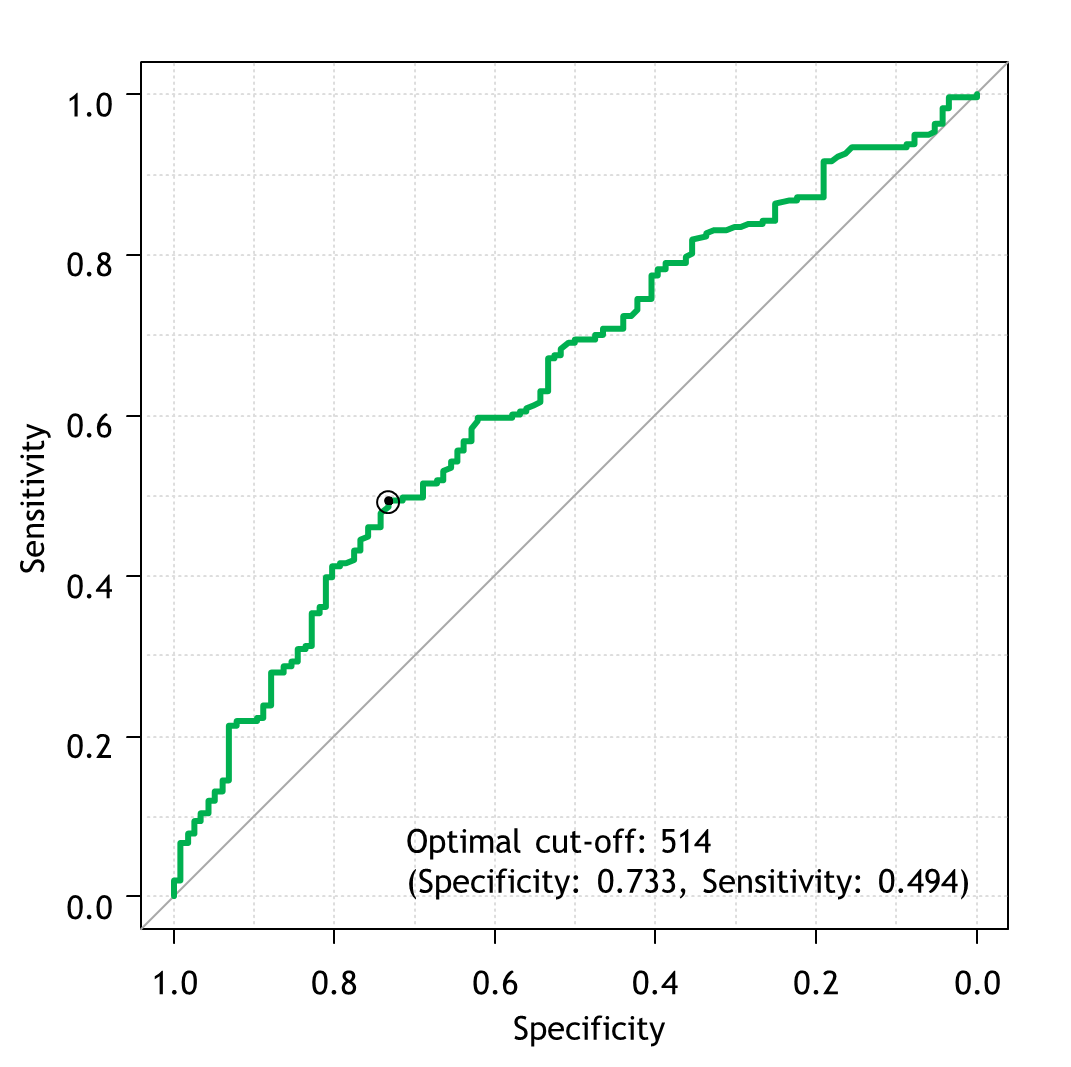


(C) Hb (D) LDH


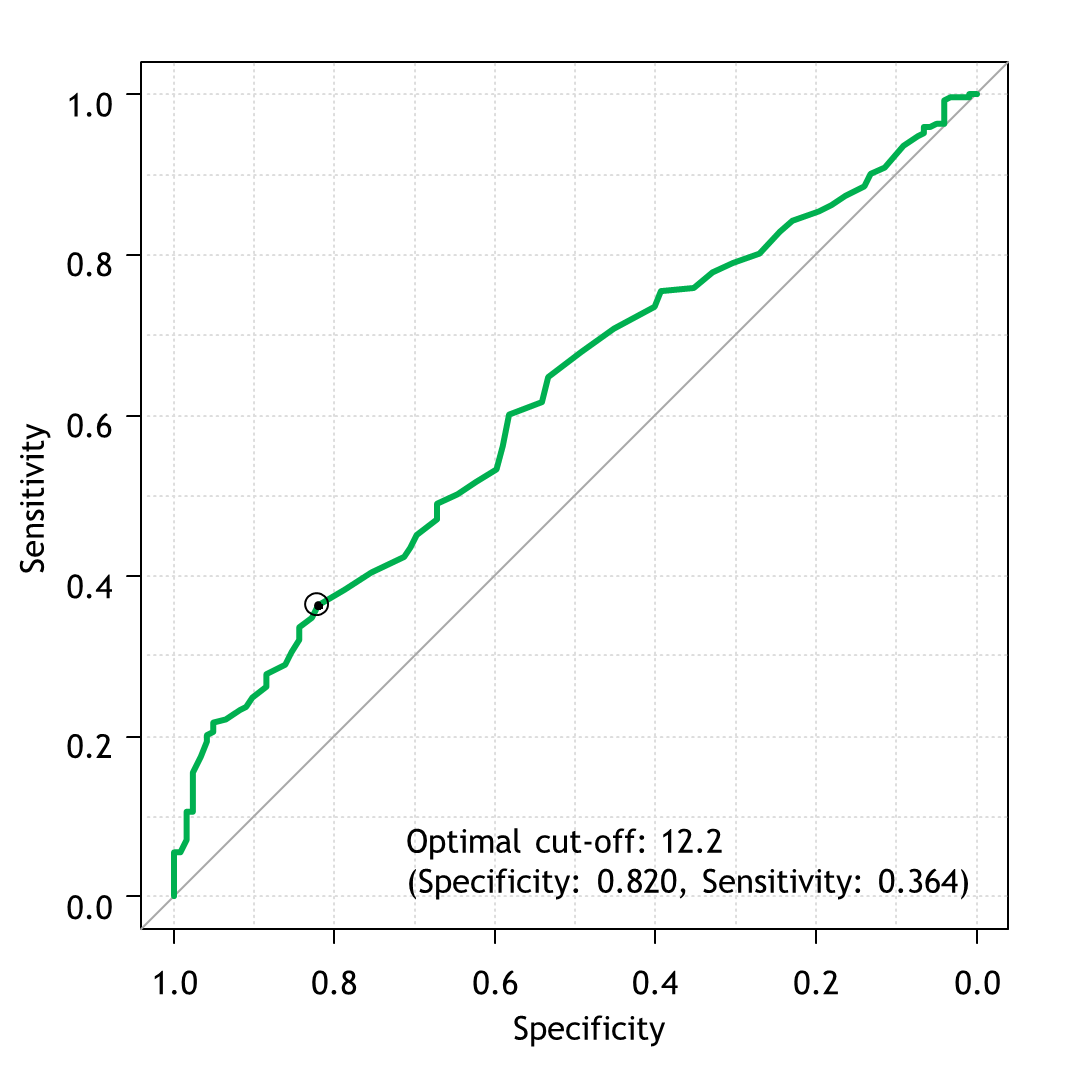

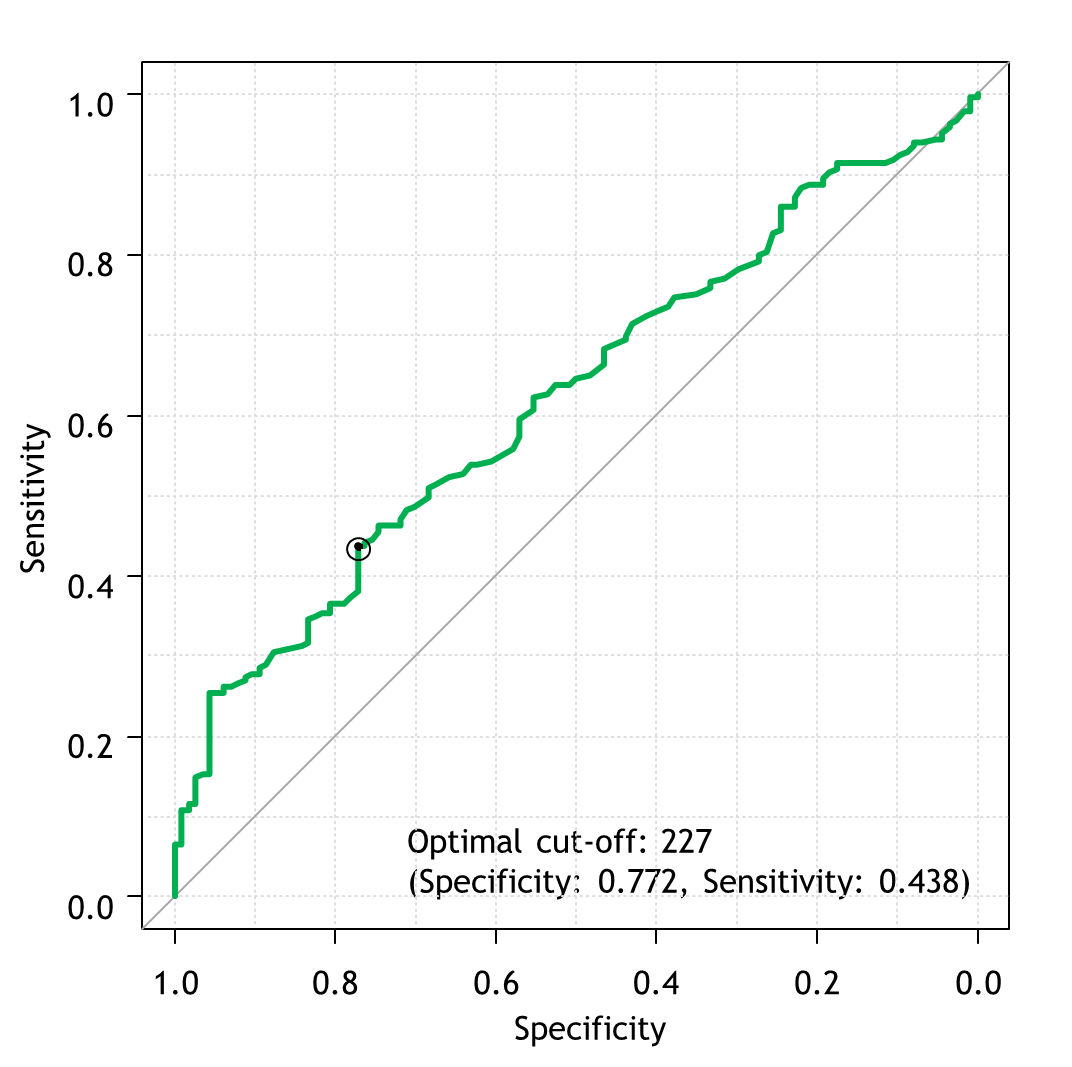


| Supplementary Table 1 Impact of possible prognostic factors on time to CRPC in entire cohort | | | | |
| --- | --- | --- | --- | --- |
|  | Univariable |  | Multivariable |  |
|  | HR (95% CI) | P value | HR (95% CI) | P value |
| Age≧75 | 1.18 (0.92-1.51) | 0.2 |  |  |
| PS≧2 | 1.34 (0.83-2.16) | 0.24 |  |  |
| PSA≧262 | 1.53 (1.20-1.96) | <0.001 | 0.88 (0.65-1.20) | 0.42 |
| Gleason score≧9 | 2.08 (1.52-2.86) | <0.001 | 1.87 (1.34-2.61) | <0.001 |
| Number of bone metastasis (EOD≧Ⅲ) | 1.88 (1.32-2.69) | <0.001 | 0.92 (0.65-1.29) | 0.63 |
| ALP≧514 | 2.27 (1.75-2.94) | <0.001 | 1.58 (1.16-2.14) | 0.003 |
| LDH≧227 | 2.30 (1.78-2.97) | <0.001 | 1.72 (1.27-2.32) | <0.001 |
| Hb<12.2 | 2.57 (1.97-3.35) | <0.001 | 1.73 (1.28-2.36) | <0.001 |
| Lung metastasis | 0.54 (0.39-0.73) | <0.001 | 0.66 (0.46-0.95) | 0.02 |
| Liver metastasis | 1.81 (1.03-3.16) | 0.04 | 2.06 (1.07-3.96) | 0.03 |
| LHRH agonist vs. antagonist | 0.99 (0.75-1.30) | 0.95 |  |  |
| NSAA vs. docetaxel | 1.58 (1.18-2.12) | 0.002 | 1.48 (1.07-3.96) | 0.03 |
| HR: Hazard Ratio, CI: Confidential Intervals, PS: Performance Status, PSA: Prostate-Specific Antigen,  EOD: Extent of Disease, ALP: Alkaline Phosphatase, LDH: Lactate Dehydrogenase, Hb: hemoglobin, LHRH: Luteinizing Hormone Releasing Hormone, NSAA: Nonsteroidal Antiandrogen  ※EODⅠ: 1 to 5 lesions, Ⅱ: 6 to 20 lesions, Ⅲ: more than 20 but less than EOD Ⅳ, Ⅳ: generalized uptake, super scan or more than 75% of axial skeleton | | | | |
|  |  |  |  |  |

| Supplementary Table 2. Differential impact of possible prognostic factors on time to CRPC between NSAA+ADT and DOC+ADT | | | | | | | | |
| --- | --- | --- | --- | --- | --- | --- | --- | --- |
|  | Docetaxel | | | | NSAA | | | |
|  | Univariable |  | Multivariable |  | Univariable |  | Multivariable |  |
|  | HR (95% CI) | P value | HR (95% CI) | P value | HR (95% CI) | P value | HR (95% CI) | P value |
| Age≧75 | 1.41 (0.81-2.47) | 0.22 |  |  | 1.01 (0.76-1.34) | 0.95 |  |  |
| PS≧2 | 2.32 (0.56-9.69) | 0.25 |  |  | 1.15 (0.69-1.92) | 0.59 |  |  |
| PSA≧262 | 2.27 (1.33-3.88) | 0.003 | 1.19 (0.64-2.21) | 0.59 | 1.35 (1.02-1.79) | 0.04 | 0.82 (0.58-1.17) | 0.27 |
| Gleason score≧9 | 1.44 (0.79-2.64) | 0.23 | 1.13 (0.59-2.18) | 0.71 | 2.48 (1.71-3.59) | <0.001 | 2.39 (1.62-3.53) | <0.001 |
| Number of bone metastasis (EOD≧Ⅲ) | 2.43 (1.41-4.18) | 0.001 | 1.07 (0.50-2.29) | 0.87 | 1.47 (1.11-1.94) | 0.007 | 0.83 (0.57-1.22) | 0.35 |
| ALP≧514 | 2.82 (1.65-4.82) | <0.001 | 2.19 (1.25-3.85) | 0.006 | 2.08 (1.55-2.80) | <0.001 | 1.41(1.00-2.00) | 0.05 |
| LDH≧227 | 2.49 (1.42-4.36) | 0.001 | 1.37 (0.74-2.55) | 0.31 | 2.15 (1.61-2.88) | <0.001 | 2.11 (1.51-2.95) | <0.001 |
| Hb<12.2 | 3.15 (1.73-5.76) | <0.001 | 3.17 (1.64-6.11) | <0.001 | 2.33 (1.73-3.13) | <0.001 | 1.78 (1.27-2.51) | <0.001 |
| Lung metastasis | 0.41 (0.21-0.82) | 0.01 | 0.33 (0.16-0.67) | 0.002 | 0.59 (0.41-0.83) | 0.003 | 0.83 (0.54-1.27) | 0.38 |
| Liver metastasis | 1.14 (0.36-3.66) | 0.82 |  |  | 2.41 (1.26-4.59) | 0.007 | 2.07 (0.95-4.51) | 0.07 |
| LHRH agonist vs. antagonist | 1.07 (0.60-1.93) | 0.81 |  |  | 0.89 (0.65-1.22) | 0.47 |  |  |
| HR: Hazard Ratio, CI: Confidential Intervals, PS: Performance Status, PSA: Prostate-Specific Antigen,  EOD: Extent of Disease, ALP: Alkaline Phosphatase, LDH: Lactate Dehydrogenase, Hb: hemoglobin, LHRH: Luteinizing Hormone Releasing Hormone, NSAA: Nonsteroidal Antiandrogen  ※EODⅠ: 1 to 5 lesions, Ⅱ: 6 to 20 lesions, Ⅲ: more than 20 but less than EOD Ⅳ, Ⅳ: generalized uptake, super scan or more than 75% of axial skeleton | | | | | | | | |
